# Comparison of the triglyceride glucose index and blood leukocyte indices as predictors of metabolic syndrome in healthy Chinese population

**DOI:** 10.1038/s41598-021-89494-9

**Published:** 2021-05-11

**Authors:** Hai-Yan Lin, Xiu-Juan Zhang, Yu-Mei Liu, Ling-Yun Geng, Li-Ying Guan, Xiao-Hong Li

**Affiliations:** Health Management Center, Shandong Provincial Hospital Affiliated to Shandong First Medical University, Jinan, 250021 Shandong China

**Keywords:** Predictive markers, Endocrine system and metabolic diseases

## Abstract

Triglyceride glucose (TyG) index and inflammatory markers are reported to have a positive association with metabolic syndrome (MetS). However, no previous study has assessed the value of TyG index and inflammatory markers as predictors of metabolic syndrome in the same study. This study looks at the comparison of the triglyceride index and blood leukocyte indices as predictors of metabolic syndrome in the Chinese population. The study cohort involved 1542 Chinese population without metabolic syndrome. The subjects underwent comprehensive routine health examination in 2011 and returned for a follow-up examination in 2016. Metabolic syndrome was defined according to Chinese Diabetes Society criteria, using body mass index for the replacement of waist circumference. TyG index, total leukocytes, neutrophils, lymphocytes, and neutrophil-to-lymphocyte ratio (NLR) were measured. Adjust d logistic models were used to assess the relationship between TyG index, blood leukocyte indices, and incident MetS. Receiver operating characteristic (ROC) curves were performed to determine the predictive value of TyG index and blood leukocyte indices for MetS. Results from multivariate logistic regression analysis showed that, in the adjusted model, the subjects with the highest quartile of TyG index and neutrophils had a 3.894- and 1.663-fold increased incidence of MetS (*P* < 0.0001 and *P* = 0.027), respectively. No significant association was observed between total leukocytes, lymphocytes, NLR with incident MetS. ROC analysis showed that the AUC of TyG index and neutrophils were 0.674 and 0.568 for incident MetS, respectively. TyG index rather than blood leukocyte indices may have the strongest predictive value in MetS development over a 5-year period.

## Introduction

Metabolic syndrome (MetS), a cluster of metabolic abnormalities characterized by obesity, hypertension, dyslipidemia, and glucose dysregulation, is associated with the risk of diabetes, cardiovascular disease, and overall mortality^1,2^. Insulin resistance plays a major role in the progression of Metabolic syndrome^3^. Chronic inflammation and oxidative stress also have been implicated in the underlying pathogenesis^4,5^.

The triglyceride glucose (TyG) index, which is calculated from fasting measurements of triglyceride and glucose, has been suggested as a reliable surrogate marker of IR^6–8^. Previous studies have found that a high baseline TyG index was related to the development of diabetes and coronary atherosclerosis^9–11^. To our knowledge, although a cross-sectional study has recently shown that TyG index was positively correlated with MetS^12^, the relationship between TyG and MetS development has not been evaluated in the longitudinal study.

Leukocytes are common, inexpensive, and broadly utilized marker of inflammation. Numerous studies have demonstrated increased total leukocytes, neutrophils, lymphocytes, and neutrophil to lymphocyte ratio (NLR) were significantly associated with MetS^13–16^. However, conclusions from some studies are inconsistent^15,17,18^. For example, Meng et al.^15^ reported that total leukocytes, neutrophils, and lymphocytes, but not NLR were associated with MetS, whereas Vahit et al.^17^ and Liu et al.^18^ reported that NLR had a significant association with MetS. However, compared with the control group, there were no changes in the level of total leukocytes and neutrophils except lymphocytes were significantly higher in the MetS group. Therefore, it is worthwhile to further evaluate the temporal association between the blood leukocyte indices and the risk of MetS. In addition, although the blood leukocyte indices and TyG index are associated with MetS, few studies have compared their predictive values in MetS development within one population.

Therefore, in this prospective study, as a primary outcome, we aimed to analyze the potential predictor value of the blood leukocyte indices in MetS development. Furthermore, the secondary outcome of this study was to compare the clinical utility of TyG index and the blood leukocyte indices in predicting incident MetS among healthy Chinese population during the 5 years of the follow-up period.

## Methods

### Study subjects

The study comprised 2585 Chinese population who had undergone routine health examination at Health Management Center of Shandong Provincial Hospital affiliated to Shandong First Medical University, China in 2011 and who had returned for a follow-up examination in 2016. Among them, the individuals (n = 628) who had an acute inflammatory disease or recent infection, renal dysfunction, liver problems, or all types of cancer, and those who (n = 241) with missing data on the questionnaire or smoking history questionnaire, anthropometric measurements, or biochemical parameters were excluded. In addition, subjects (n = 178) with MetS were further excluded at baseline. Therefore, a total of 1542 subjects (1056 men and 486 women) with a mean age of 44.8 ± 12.6 years (range: 22–85 years) remained. The study was approved by the institutional review board of Shandong Provincial Hospital affiliated to Shandong First Medical University. Written informed consents were obtained from all subjects prior to the health check-up when they visited the Health Management Center.

### Study measurement

The physical examination comprised blood pressure (BP) and anthropometric measurements, including height, weight, and body mass index (BMI). BMI was calculated as weight (kg) divided by height (m)^2^. The BP was measured by well-trained nurses two times in the left arm of seated participants using an automated electronic device (OMRON model HEM-752 Fuzzy, Omron Company, Dalian, China) after 5-min sitting. The average of the two readings was calculated to determine the reported BP. Blood samples were collected from subjects who had fasted for ≥ 12 h. The levels of fasting blood glucose (FBG), lipids including triglyceride (TG), total cholesterol (CH), high-density lipoprotein cholesterol (HDL-C), low-density lipoprotein cholesterol (LDL-C) were measured using an Olympus AU5400 system. The TyG index was calculated as ln[fasting triglycerides (mg/dl) x fasting glucose (mg/dl)/2]^19^. Total leukocytes, including neutrophils and lymphocytes count analysis, were performed in the hematology laboratory of our hospital, and NLR values were calculated. Smoking habit was defined by having ≥ 5 cigarettes per day.

According to the guidelines for Type 2 Diabetes in China by Diabetes Branch of the Chinese Medical Association^20^, using BMI for the replacement of waist circumference, the MetS was defined as having any three or more of the following factors: (a) overweight and/or obesity: BMI ≥ 25.0 kg/m^2^; (b) raised FPG: ≥ 6.1 mmol/L or 2-h plasma glucose (2hPG) level ≥ 7.8 mmol/L or previously diagnosed as type 2 diabetes and taking anti glycemic medication; (c) raised BP: ≥ 130/85 mmHg, or previously diagnosed as hypertension and taking antihypertensive medication; (d) raised TG: ≥ 1.7 mmol/L; and/or (e) reduced HDL-C: < 1.04 mmol/L.

### Statistical analysis

SPSS19.0 was used for data analysis, and PASS11 was used to estimate the sample size and analyze the statistical power of the results. A value of less than 0.05 was deemed statistically significant. The distribution of the different variables was examined for normality by the Kolmogotov-Smirnov test. Categorical variables were expressed in percentages and continuous variables in mean (SD) or geometric mean (95% confidence interval).

Between-group differences with respect to categorical variables were assessed using a Chi-square test. Between-group differences with respect to continuous variables of normal distribution were assessed using Student test or one-way ANOVA, and continuous variables of non-normal distribution were assessed using Mann–Whitney U test or Kruskal–Wallis test.

Multivariate logistic regression analysis was used to calculate the odds ratios (ORs) and 95% confidence intervals (CI) of incident MetS for each investigated indicator quartile compared with the lowest quartile, with adjustment for baseline age, sex, smoking status (yes, no), BMI, SBP, DBP. The age, BMI, SBP, DBP were treated as continuous variables. A receiver operating characteristic (ROC) curve analysis, which was quantified by the area under the ROC curve (AUC), was used to assess the value of indicators for predicting incident MetS. The area under ROC curve was compared by the nonparametric *Z* test. Youden’s index (sensitivity + specificity  − 1) was used to determine the optimal cutoff point of each indicator.

All above methods were carried out in accordance with relevant guidelines and regulations.

### Ethical approval

The study was approved by the institutional review board of Shandong Provincial Hospital affiliated to Shandong First Medical University.

## Results

A total of 1542 metabolic syndrome-free participants at baseline were enrolled in this study. Of these, 179 (11.4%) subjects who developed MetS during the 5 years follow-up period were categorized as positive for MetS (MS), while 1363 (88.6%) subjects free of MetS were categorized as NMS. The baseline demographic characteristics and laboratory parameters of the population are shown in Table 1. A significant between-group difference in TyG index was observed in the subjects with MetS compared to subjects free of MetS (8.75 (8.68, 8.82) vs 8.45 (8.43, 8.48); *P* < 0.0001). Compared with subjects free of MetS, those with MetS were tend to have higher levels of total leukocytes, neutrophils, and lymphocytes. In addition, a higher BMI, SBP, DBP, CH, TG, LDL-C, and fasting blood glucose, the proportion of male subjects smokers, along with a lower level of HDL-C were observed in subjects with MetS (*P* < 0.01 for all). No significant differences were observed on NLR (*P* = 0.736) and age (*P* = 0.655) between MS and NMS groups.

**Table 1:** Participant baseline demographic characteristics and laboratory parameters by metabolic syndrome status (n = 1542).

Characteristic	NMS	MS	*P-*Value
Subjects	1363	179	
Age (years)	44.9 (44.2, 45.6)	45.0 (44.3, 46.7)	0.655
Gender (male-n-%)	902(67.0%)	154 (86.0%)	< 0.0001
Smoking (n-%)	232 (17.2%)	44 (24.6%)	0.01
Body mass index (kg/m^2^)	24.0 (23.9, 24.2)	26.2 (25.8, 26.6)	< 0.0001
Systolic blood pressure (mmHg)	119.5 (118.7, 120.4)	125.8 (123.7, 128.0)	< 0.0001
Diastolic blood pressure (mmHg)	70.2 (69.6, 70.7)	75.4 (73.8, 77.0)	< 0.0001
Leukocytes (× 10^9^/L)	5.21 (6.14, 6.28)	6.60 (6.39 6.81)	0.001
Neutrophils (× 10^9^/L)	3.39 (3.33, 3.44)	3.60 (3.46, 3.75)	0.003
Lymphocytes (× 10^9^/L)	2.25 (2.22, 2.28)	2.39 (2.30, 2.49)	0.003
NLR	1.59 (1.56, 1.62)	1.57 (1.50, 1.65)	0.736
High-density lipoprotein (mmol/L)	1.38 (1.36, 1.39)	1.22 (1.19, 1.25)	< 0.0001
Low-density lipoprotein (mmol/L)	3.28 ± 0.02	3.44 ± 0.82	< 0.0001
Total cholesterol (mmol/L)	5.13 ± 0.25	5.21 ± 0.78	< 0.0001
Triglyceride (mmol/L)	1.24 (1.20, 1.28)	1.62 (1.48, 1.75)	< 0.0001
Fasting blood glucose (mmol/L)	5.41 (5.37, 5.45)	5.53 (5.43, 5.64)	< 0.0001
TyG index	8.45 (8.43, 8.48)	8.75 (8.68, 8.82)	< 0.0001

To determine independent variables for the incidence of MetS, multivariate logistic regression analysis was performed and results showed in Table 2. Subjects with the higher TyG index, total leukocytes, neutrophils, and lymphocytes significantly increased the incidence of MetS (*P* < 0.05 for all). After adjusting for age, sex, and smoking habits, total leukocytes and lymphocytes were no longer independent predictors of MetS (model 1), whereas the associations between TyG index, neutrophils, and incident MetS had no change, and continued to maintain even after further adjustments were made for BMI and blood pressure levels (model 2). In the final model, the subjects with the highest quartile of TyG index and neutrophils had a 3.894- and 1.663-fold increased incidence of MetS (*P* < 0.0001 and *P* = 0.027), respectively.

**Table 2:** Associations of TyG index, inflammatory markers and risk of metabolic syndrome incidence (odds rations and 95% confidence intervals).

	Quartiles				*P* for trend
	Q1	Q2	Q3	Q4	
**TyG index**					
Range	≤ 8.14	8.15–8.48	8.49–8.81	> 8.82	
MS cases/total number of each quartile (%)	12/386	39/387	55/394	73/375	
No adjusted	1	3.496 (1.799–6.780)	5.057 (2.662–9.604)	7.534 (4.016–14.132)	< 0.0001
Model 1	1	3.015 (1.542–5.896)	4.127 (2.146–7.936)	5.924 (3.113–11.275)	< 0.0001
Model 2	1	2.316 (1.169–4.588)	2.713 (1.389–5.300)	3.894 (2.011–7.537)	< 0.0001
**Total leukocytes**					
Range	≤ 5.30	5.31–6.12	6.13–7.03	> 7.04	
MS cases/total number of each quartile (%)	32/389	43/384	45/385	59/384	
No adjusted	1	1.407 (0.870–2.276)	1.477 (0.916–2.379)	2.025 (1.284–3.195)	0.021
Model 1	1	1.252 (0.769–2.038)	1.255 (0.773–2.037)	1.692 (1.064–2.689)	0.147
Model 2	1	1.187 (0.721–1.956)	1.102 (0.671–1.809)	1.298 (0.806–2.092)	0.730
**Neutrophils**					
Range	≤ 2.72	2.73–3.28	3.28—3.95	> 3.96	
MS cases/total number of each quartile (%)	31/387	50/384	36/387	62/384	
No adjusted	1	1.719 (1.072–2.757)	1.178 (0.713–1.946)	2.211 (1.400–3.491)	0.002
Model 1	1	1.707 (1.059–2.752)	1.044 (0.628–1.735)	2.090 (1.317–3.317)	0.002
Model 2	1	1.606 (0.985–2.618)	0.971 (0.579–1.630)	1.663 (1.034–2.672)	0.027
**Lymphocytes**					
Range	≤ 1.85	1.86–2.20	2.21–2.62	> 2.63	
MS cases/total number of each quartile (%)	33/390	46/383	40/387	60/382	
No adjusted	1	1.477 (0.922–2.366)	1.247 (0.769–2.023)	2.016 (1.284–3.164)	0.015
Model 1	1	1.378 (0.854–2.224)	1.066 (0.650–1.750)	1.663 (1.048–2.639)	0.089
Model 2	1	1.301 (0.796–2.126)	0.959 (0.578–1.592)	1.283 (0.797–2.064)	0.421
**NLR**					
Range	≤ 1.17	1.18–1.47	1.48–1.88	> 1.89	
MS cases/total number of each quartile (%)	42/390	48/388	45/386	44/378	
No adjusted	1	1.170 (0.753–1.817)	1.093 (0.700–1.708)	1.092 (0.697–1.710)	0.762
Model 1	1	1.155 (0.740–1.801)	1.102 (0.702–1.730)	1.186 (0.752–1.869)	0.888
Model 2	1	1.148 (0.728–1.810)	1.094 (0.689–1.736)	1.174 (0.736–1.873)	0.908

Data are expressed as ORs (95% CI). Model 1: adjusted for age, sex, smoking; model 2: Model 1 adjusted further for body mass index, blood pressure.

**Figure 1:**
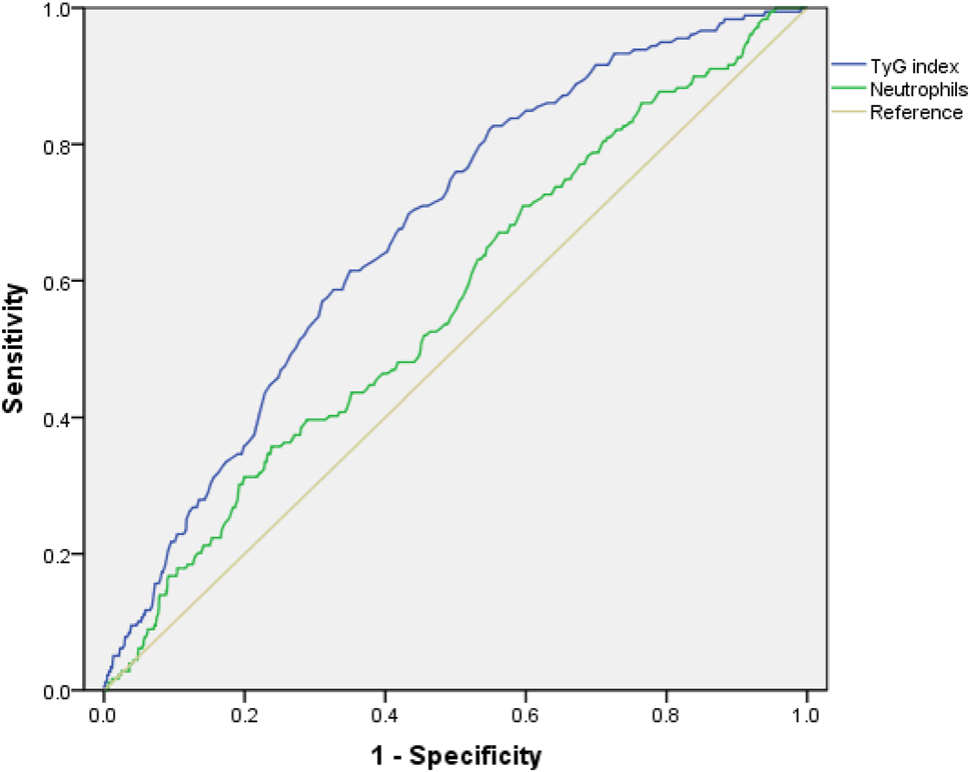
Receiver operating characteristic (ROC) curves of TyG index and neutrophils in predicting incident metabolic syndrome.

The accuracy of TyG index and neutrophils and their sensitivity and specificity in predicting incident MetS were compared and the results showed in Fig. 1 and Table 3. The AUC of TyG index and neutrophils were 0.674 and 0.568 for incident MetS, respectively. Further *Z* test was performed to compare the area under ROC curve, results showed that the difference was statistically significant (Z = 3.56 P < 0.05). The statistical power for two ROC curves was 0.98 with *β* = 0.0165. The optimal cutoff value of TyG index, neutrophils based on Youden's index in predicting incident MetS was 8.52, 3.26 × 10^9^/L, with a sensitivity and specificity of 70.4% and 55.9%, 55.3%, and 50.3%, respectively.

**Table 3:** Areas under the ROC Curve (AUC), sensitivity and specificity by the optimized cutoff points for TyG index and neutrophils in predicting metabolic syndrome.

	AUC (95%CI)	Cutoff	Sensitivity (%)	Specificity (%)
TyG index	0.674 (0.635, 0.713)	8.52	70.4	55.9
Neutrophils (× 10^9^/L)	0.586 (0.560, 0.612)	3.26	55.3	50.3

## Discussion

The current study examined associations between TyG index, blood leukocyte indices (total leukocytes, neutrophils, lymphocytes, NLR), and incident MetS, moreover compared their predicting values in MetS development in a normal check-up adult population during the 5 years of follow-up period. The results have demonstrated that, of the blood leukocyte indices, although total leukocytes, neutrophils, and lymphocytes were each associated with the incident MetS, neutrophils were the most strongly associated with MetS after adjusting for potential confounding factors (including age, sex, BMI, smoking status, and blood pressure). The most importantly, a ROC curve analysis in this study indicated that TyG index was more useful for predicting individuals who were most likely to develop MetS than neutrophils. To our knowledge, this study is the first prospective study to assess the predicting value of TyG index and blood leukocyte indices in incident MetS within one healthy population.

TyG index has been reported to be closely correlated with insulin resistance, the presence of coronary artery complications in type 2 diabetes and healthy adults. For example, previous cross-sectional studies suggested that TyG index was a useful marker for early identification of insulin resistance individuals^6^, and the predictive value of the TyG index for insulin resistance was even better than that of the HOMA-IR^21,22^. A 4-year retrospective longitudinal study in non-diabetic adults indicated that high baseline TyG index was related to diabetes development^9^. Moreover, it was confirmed that a high TyG index was associated with an increased risk of microalbuminuria and cerebrovascular disease among type 2 diabetes patients^23^. Previous studies have shown that TyG index was associated with coronary atherosclerosis^10,11^, and was also a useful marker for predicting subclinical coronary in healthy individuals with a low CV risk burden^24^. Results from the most current cross-sectional study showed that TyG index increased with the number of MetS components^12^. In our present longitudinal study, it is further confirmed that a high baseline TyG index is associated with the development of MetS in the healthy population.

Total leukocytes, neutrophils, and lymphocytes are common, inexpensive, and broadly utilized markers of inflammation. They activate major cell types involved in acute and chronic inflammation. Similar to previous studies^15^, increased total leukocytes, neutrophils, and lymphocytes were observed in the subjects of developed MetS in the present study. Previous studies presented that systemic inflammatory markers were associated with age, gender, smoking history, body weight, and disease of hypertension^25–29^. Therefore, confounding factors including age, sex, smoking status, BMI, and blood pressure were taken into account in our analysis. After adjusting for the multiple potential confounding factors, the associations between total leukocytes and lymphocytes with incident MetS no longer existed. However, neutrophils continued to have a strong association with incident MetS. These findings are consistent with previous findings that neutrophils is a useful biomarker of inflammation in nascent MetS^30^.

The neutrophil-to-lymphocyte ratio (NLR), calculated by dividing the neutrophil by the lymphocyte, has been found to be a reliable indicator of inflammation and correlates with the presence and severity of MetS^18,31^. It is interesting that the previous cross-sectional studies have reported a positive association between NLR and MetS, in contrast, our study did not find this association. A most recent study showing a no-significant increase in NLR in patients with nascent MetS^30^, which may explain why our findings did not show a positive correlation between NLR and the development of MetS.

It is noteworthy that the present study provides beneficial data for comparing the value of TyG index and leukocyte indices in predicting incident MetS in one report. A high AUC of the TyG index was expected, as the TyG index includes triglyceride and glucose, the main components of the metabolic syndrome. As determined based on the AUC of the ROC curve and a further *Z* test, TyG index had a greater predictive power for incident MetS than neutrophils.

The present study showed the optimal TyG index cutoff for incident MetS in Chinese adults was 8.52. Shin et al^12^ reported the optimal cutoff of TyG index to be 8.81 for MetS in middle-aged and older Korean populations. Based on the findings, it is suggested there might be a standardized cut-off value for TyG index in estimating the development of MetS, as well as initial evaluation^12^. Due to the importance of early lifestyle intervention in the treatment of MetS, elevated TyG index levels can provide a significant clue for the assessment of the high-risk MetS population. Given the variability of triglyceride levels according to ethnicity, further studies are needed to assess the TyG index in other populations.

There are several limitations of the present study that need to be noted. First, the objects of study and analysis were those who visited the health management center and were annually follow-up examination, therefore, the findings of this study might not be representative of the general population. Second, the diagnostic criteria for MetS in this study were not the most commonly used International Diabetes Federation and the National Cholesterol Education Adult Treatment Panel III-R, but the Diabetes Branch of the Chinese Medical Association based on the study of MetS in China. However, which is more consistent with the Chinese characteristics. Third, instead of waist circumference, BMI was used in the definition of MetS due to a lack of corresponding data. This lack of data might have led to a miss estimation of the actual prevalence of MetS. Fourth, our study population was relatively small, therefore the statistical power may be limited due to the small number of incident cases.

In conclusion, compared with the total leukocytes, lymphocytes, and NLR, neutrophils appear to be a robust cost-effective marker of inflammation in predicting the incidence of MetS in healthy people. Furthermore, TyG index appears to be superior to the neutrophils in predicting the risk of incident MetS in this group. Additional large and long-term prospective studies are required to establish the role of TyG index in the MetS development.
